# Unveiling the nature of interaction between semantics and phonology in lexical access based on multilayer networks

**DOI:** 10.1038/s41598-021-93925-y

**Published:** 2021-07-14

**Authors:** Orr Levy, Yoed N. Kenett, Orr Oxenberg, Nichol Castro, Simon De Deyne, Michael S. Vitevitch, Shlomo Havlin

**Affiliations:** 1 Department of Physics, Bar-Ilan University, Ramat-Gan, 52900 Israel; 2 Faculty of Industrial Engineering and Management, Technion—Israel Institute of Technology, 3200003 Haifa, Israel; 3 Department of Communicative Disorders and Sciences, University at Buffalo, Buffalo, NY 14214 USA; 4 School of Psychological Sciences, University of Melbourne, Melbourne, VIC 2010 Australia; 5 Department of Psychology, University of Kansas, Lawrence, KS 66045 USA

**Keywords:** Psychology, Human behaviour, Physics, Statistical physics, thermodynamics and nonlinear dynamics, Complex networks

## Abstract

An essential aspect of human communication is the ability to access and retrieve information from ones’ ‘mental lexicon’. This lexical access activates phonological and semantic components of concepts, yet the question whether and how these two components relate to each other remains widely debated. We harness tools from network science to construct a large-scale linguistic multilayer network comprising of phonological and semantic layers. We find that the links in the two layers are highly similar to each other and that adding information from one layer to the other increases efficiency by decreasing the network overall distances, but specifically affecting shorter distances. Finally, we show how a multilayer architecture demonstrates the highest efficiency, and how this efficiency relates to weak semantic relations between cue words in the network. Thus, investigating the interaction between the layers and the unique benefit of a linguistic multilayer architecture allows us to quantify theoretical cognitive models of lexical access.

## Introduction

Language is a core cognitive capacity in human communication and interaction. It involves many different linguistic components, including phonology, morphology, syntax, semantics, and pragmatics^1^. Across the complex process that enables efficient communication via language, one crucial question is: How do humans comprehend and produce meaningful linguistic output? A critical assumption regarding the realization of such communication skills, is the availability of conceptual representation in the cognitive system, e.g., the ‘mental lexicon’^2^. Classic linguistic theories assume that in order to comprehend or produce meaningful linguistic output, one needs to access and retrieve information from their mental lexicon, known as lexical access. Lexical access involves multiple processes of representation, in particular a semantic word-meaning process and a phonological word-form mapping process, that allow access and retrieval from the mental lexicon^2,3^. However, whether the relation between these two processes is serial, parallel, or interactive is still debated^3,4^. In the current study we develop a computational framework based on a multilayer network to investigate the relation and the mutual effect of phonology and semantics on each other, to directly study the relations between these two processes.

Linguistic theories on the relation between semantic and phonological components of lexical access propose either a modular, cascading, or an interactive process of these two components. The modular account argues for a detailed process between two discrete modular processes of lexical access^5^. According to this account during lexical access of a linguistic input, phonological processing takes place only after semantic processing is completed. The cascading account argues for a more relaxed modular account. According to this model, phonological processing can initiate before semantic processing is complete. Finally, the interactive model^2^ theorizes that lexical access involves an interactive spread of information across a phonological layer and a semantic layer that can influence each other. This model argues that both layers are structured as a network and that information spreads across these two networks, related to the organization of concepts across both layers and to the strength of links that connect them^3^. Thus, for example, when the concept *cat* is activated, both semantically connected concepts such as *dog* and *milk* are automatically activated, but also phonologically connected concepts such as *mat* or *hat* are activated, and a selection mechanism selects the appropriate concept.

Empirical support for the interactive model has been shown in semantically mediated phonological priming, homophone processing^6–8^, and false memory studies^9–12^. In semantically mediated phonological priming studies, it was shown that participants were quicker to respond to targets following primes that were indirectly phonologically related^6,7^. In a homophone study, it was shown that participants are more likely to accept homophone foils as members of a semantic category when the correct homophone was a typical member of that category (e.g. plain is accepted as an air vehicle^8^). In false memory studies, the recall of a word that was not part of a prior learnt list of words (critical lure; *cow* in this example) is tested when the learned list included a hybrid of semantically and phonologically related words (e.g., chill, told, warm, old, shiver, called, winter, sold, freezer, coal, snow, polled^9^). These studies show higher false memories in these hybrid lists compared to pure lists of either kind^10^. Recently, such higher false memories to hybrid lists have been argued to be related to the interactive model^11^. According to this theory, this effect is due to additive activation in both semantic and phonological networks related to the critical lure, allowing this activation to surpass a threshold for output^11^.

Thus, theoretical and empirical evidence from language and memory research demonstrate the interaction of phonological and semantic processing, highlighting the need to further elucidate the nature of their interaction. However, such theories are largely indirectly examined via behavioral studies (but see^13^). In the current study, we apply a computational network science approach to examine the interaction between phonological and semantic networks. Network science is based on graph theory, providing quantitative methods to investigate complex systems as networks^14^. Application of network science methodologies to study cognitive systems have been recently gaining recognition, with an emphasis on studying language and memory systems^15–17^. While such network applications have led to unique insights in studying separately phonology^18^ and semantics^19^, they primarily focus on one layer (phonological or semantic) at a time and do not examine the interaction between the two levels.

The multilayer network approach allows examining a network where the same set of nodes are connected differently across layers of networks^20–22^. It has been successfully applied in different domains, such as robustness of infrastructure, science of science and game-theoretic dilemmas, among many others^22^. Recently, multilayer network analysis has been applied in studying language, mainly to quantify language development and impairment^23–26^. In this line of research, the linguistic multilayer network includes four layers, roughly mapping onto layers of phonology—one layer—and semantics—three layers based on free associations, synonyms, and taxonomies^27^. Thus, while other linguistic components such as syntax and morphology can be added as additional layers when suitable data will be available, currently our work focuses on a phonological-semantic linguistic multilayer network where suitable extensive data exists.

**Figure 1:**
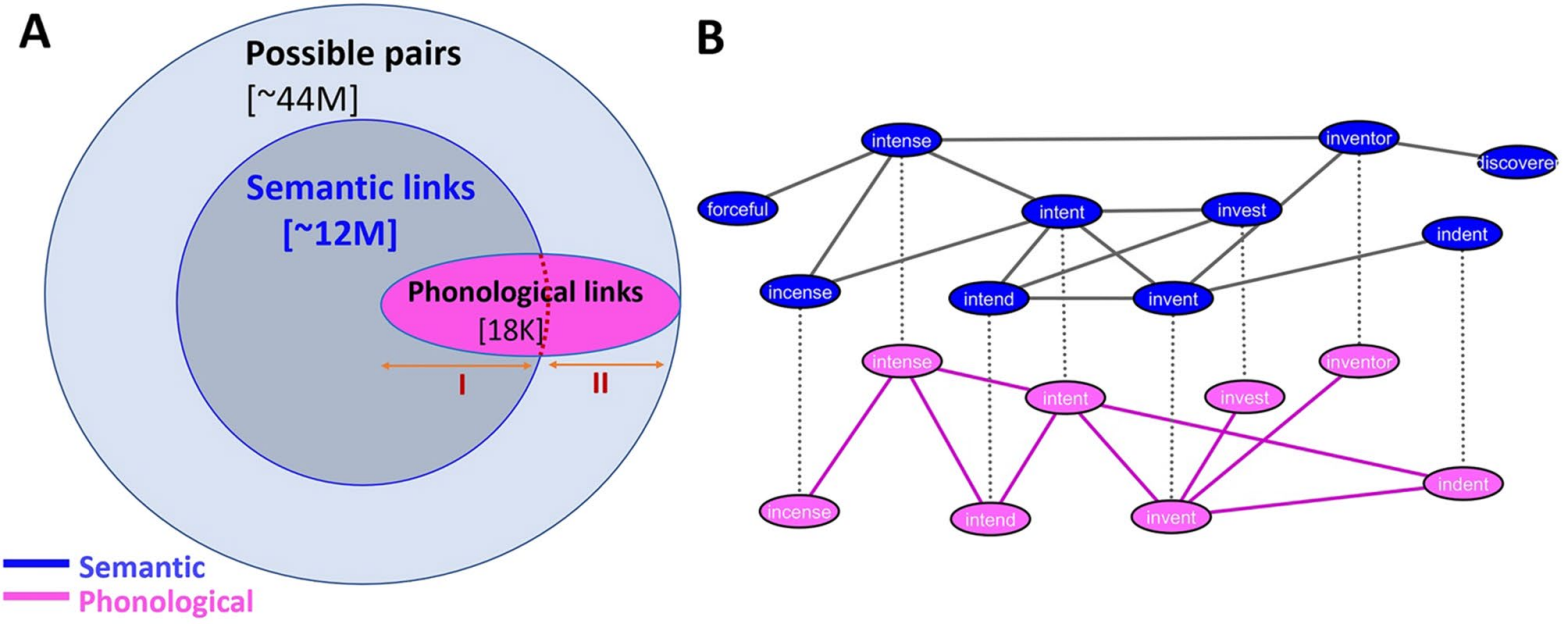
Multiplex network of semantic and phonological layers. (**A**) Illustration of the overlap across the (weighted) semantic layer and the (unweighted) phonological layer. (**B**) Illustration of the multiplex network with nodes and links across both semantic and phonological layers.

**Figure 2:**
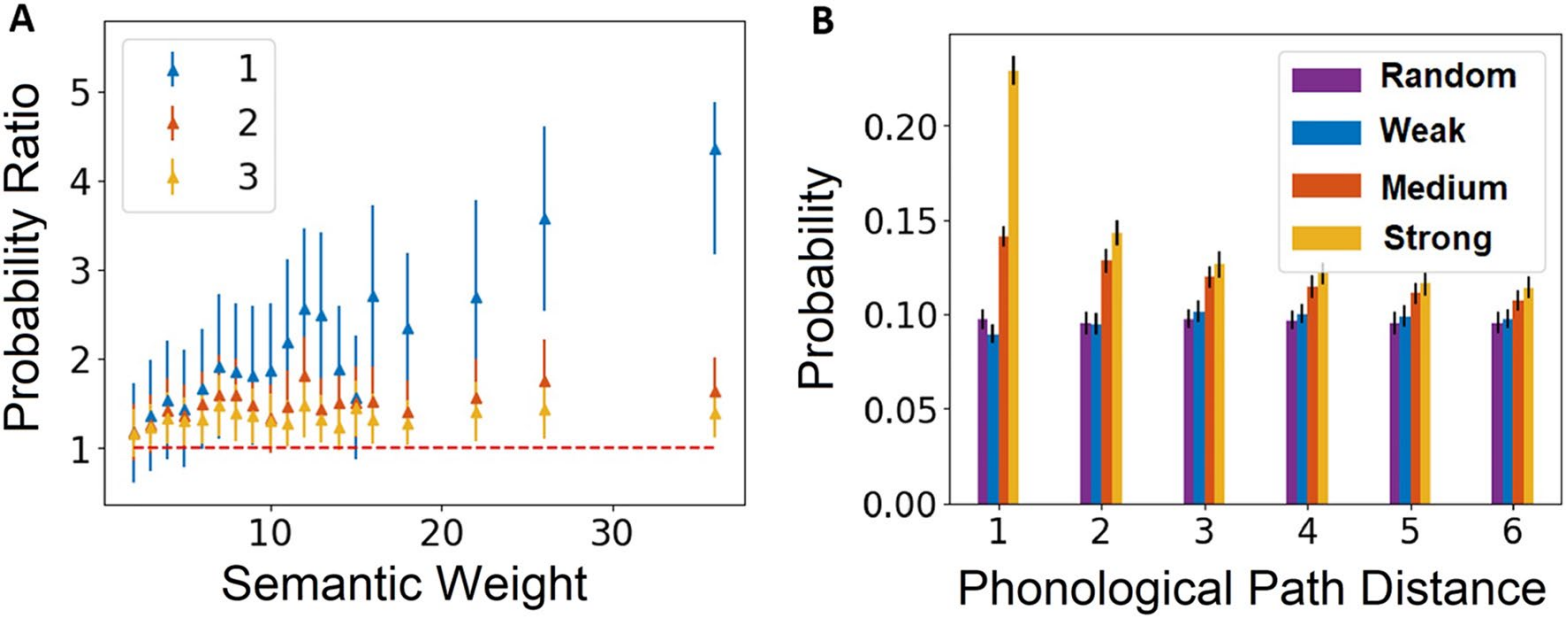
Phonological and semantic overlapping links analysis. (**A**) The overlap ratio between the number of phonological links within semantic links and the number of the overlapping shuffled phonological links within semantic links, as a function of semantic weight, for different phonological paths distances (1, 2, and 3 in the figure). Each point represents the ratio of the *average* number of overlapping phonological links (for a specific phonological path distance) divided by the average number of shuffled phonological links within the semantic window (bars denote standard error). These averages were obtained from 100 iterations per window. (**B**) Fraction of overlapping semantic links of different strength with phonological links of different phonological distances. The fraction is with respect to the phonological network. For each phonological path distance, we compared the average fraction of overlapping groups of semantic links of different strength – strong, medium, weak links and shuffled semantic links with phonological links (bars denote standard error). These averages were obtained from 100 iterations per group. Note that for phonological path distance one, the fraction of overlapping phonological linked with all semantic strengths is close to 0.5.

**Figure 3:**
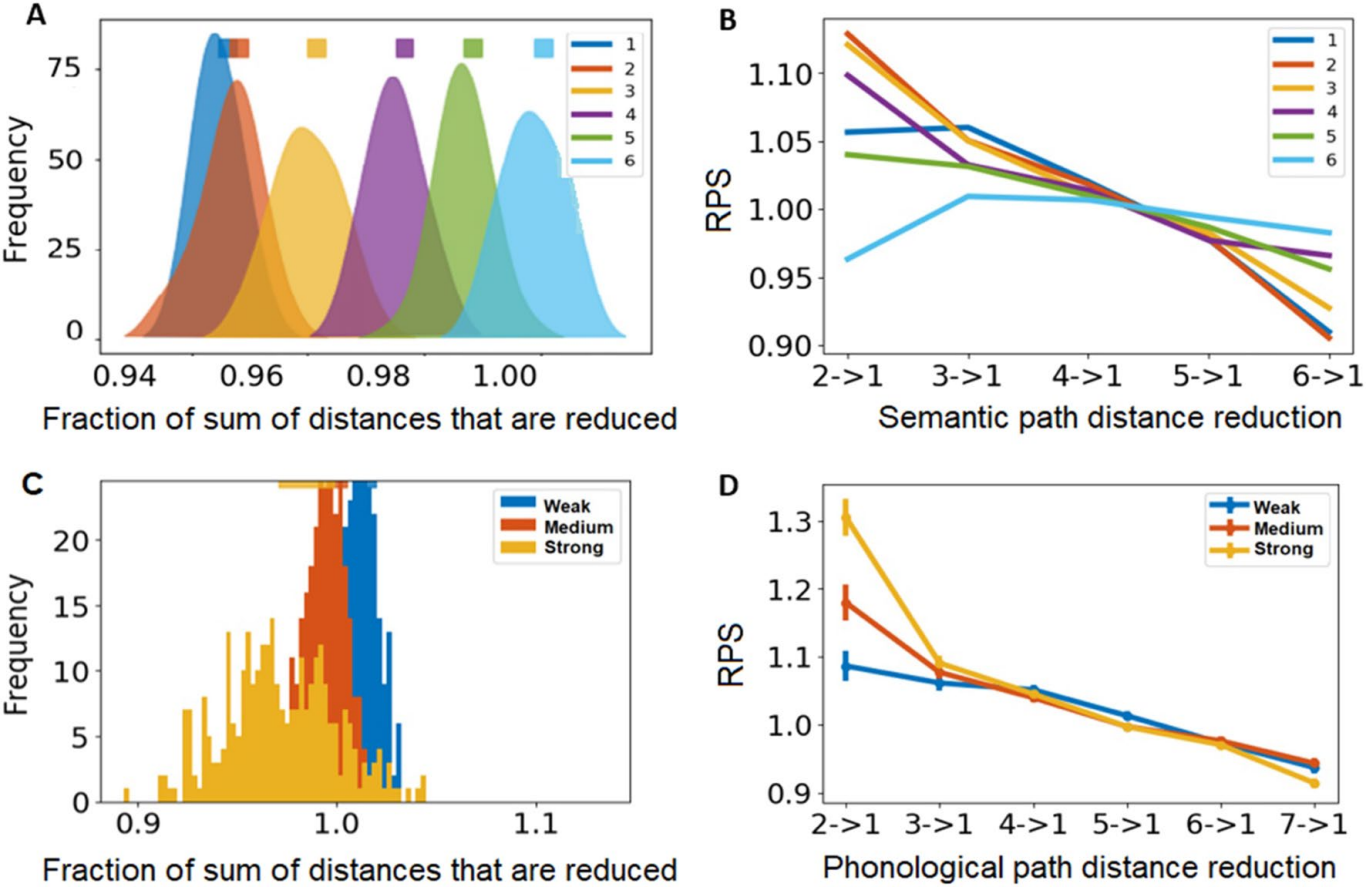
The effect of adding non-overlapping phonological/semantic links to the semantic/phonological layer. (**A**) The ratio between the sum of distances that were reduced in the semantic layer by adding non-overlapping phonological links of different distances and by adding randomly chosen links (adding random links corresponds to 1.00 in the x-axis). The smooth kernel histogram distribution is shown for 300 realizations. The rectangles represent the mean and standard deviation of the specific phonological-distance distributions. (**B**) Ratio between the number of semantic path distances that were reduced to a distance of one due to adding non-overlapping phonological links and the analogues for adding random links. The x-axis is the specific semantic path distances that were reduced. Y-axis: Ratio of Path Shortening (RPS). (**C**) The ratio between the sum of distances that were reduced in the phonological layer by adding non-overlapping semantic links from different strengths (weak, medium and strong) and by adding randomly chosen links. The distribution is shown for 300 realizations. The rectangles are colored according to the phonological path distance represents the mean and the standard deviation of the distributions. (**D**) Ratio between the number of phonological path distances that were reduced to a distance of one due to adding non-overlapping semantic links and the analogues for adding random links. The x-axis is the specific phonological path distances that were reduced. Y-axis: Ratio of Path Shortening (RPS).

**Figure 4:**
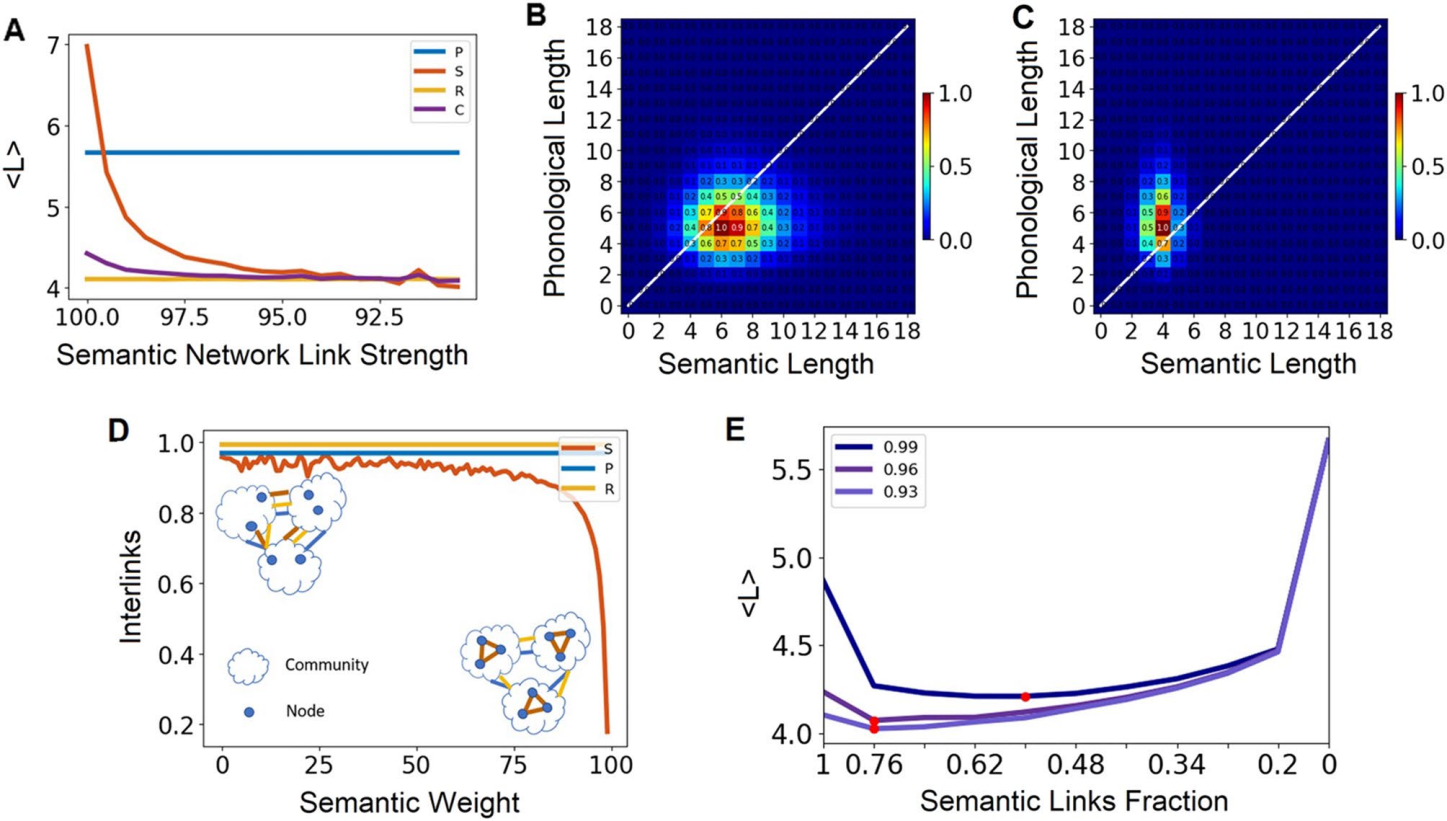
Comparing the distances of the semantic, phonological and multilayer networks as a function of the strength of the semantic links. (**A**) We compared the mean distances of the phonological network (P), the semantic filtered network (S), a multilayer composed of phonological and semantic filtered layers (M), and a random network with the same degree (R). All four networks consisted of the same set of nodes and have the same average degree. (**B**) Density plot of pairwise distances of the phonological network and the semantic network of top percentile of strongest semantic links. Note that the semantic network has typically longer distances than the phonological network. (**C**) Density plot of distances of the phonological network and the semantic network of the top 90% of strongest semantic links. Note that semantic network has typically shorter distance. (**D**) Comparison of the fraction of inter-links (inter community links) of the semantic network as a function of the semantic strength for the semantic (S), phonological (P) and random (R) networks. We found that phonological links and weak semantic links tended to be inter-community links more than strong semantic links, which explains the distance effect of the groups. (**E**) The average distance of the multilayer phonological-semantic network for different percentages of top semantic links (99%, 96% and 93%) as a function of the fraction of the semantic links. While in (**A**) we measured only the case of 50%, here we tested different fractions and we found that the minimal (optimal) average distance in the multiplex network of stronger semantic links was obtained for larger fractions of phonological links.

**Figure 5:**
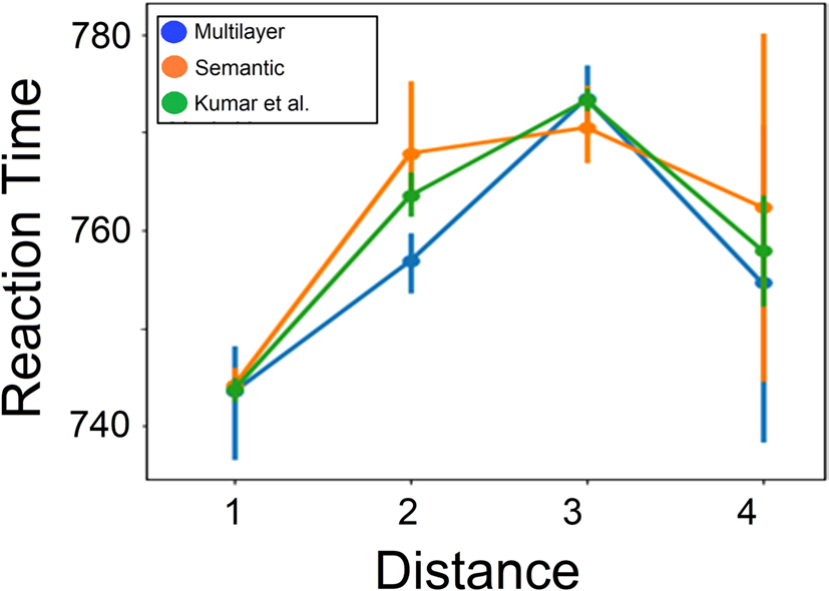
Multilayer network is more viable than the semantic network. RTs for relatedness judgments in the Kumar, Balota, and Steyvers^32^ network (Green), semantic network (Orange) and multilayer network (Blue). Error bars represent standard deviations of the average RT. The error bar of the Kumar, Balota, and Steyvers^32^ network represents the standard deviation of averages of subsets of RTs.

In the present study we apply a multilayer network approach to directly analyze and quantify the relation between phonological and semantic networks, motivated by the interactive model of language processing^3^. We do so by constructing a large-scale multilayer network comprised of empirical phonological and semantic layers (Fig. 1), for a large-scale network of about 9000 words. These words were used in a unique, multi-year big data free association data acquisition project^28^. While textual corpus based methods exist to estimate semantic networks^29^, analyzing behavior-based data such as collected via free association data is argued to better represent the ‘mental lexicon’^30,31^. First, we examine the similarity between the two layers by measuring their link overlap (Fig. 2). Next, we measure the effect of adding non-overlapping links from one layer to the other (Fig. 3). Finally, we examine the potential benefit of combining both layers as a multilayer network on lexical access, by measuring the networks’ average distances of the single layers versus the multiplex (Fig. 4). Past research has demonstrated the role of shorter distances on cognitive processing in typical and clinical populations using semantic, phonological, and phonological-semantic networks^26,31–34^. Thus, we expect to find optimal distances in our multilayer network compared to independent phonological and semantic layers. We directly test this prediction by comparing how distances in our multilayer network better captures Reaction Time data collected in a previous study^32^ (Fig. 5). Our analyses aim to elucidate the different proposed theoretical relations between phonology and semantics in lexical access (serial, parallel, or interactive).

## Materials and methods

### Data collection

Data analyzed is a subsample of a large free association data collection project in English^28^. Ethics approval was obtained from the KU Leuven ethics committee (ref. G-2014 07 017), obtained by co-author Dr. Simon De Deyne. All participants provided their informed consent to participate in this data collection study^28^. Importantly, all analysis methods we conducted in this study preserved and respected the anonymity of participants that took part in the data collection study, and were carried out in accordance with relevant guidelines and regulations by the KU Leuven Ethics Committee^28^. This online data collection project follows a similar free association data collection project previously conducted in Dutch^35^, and was approved by the KU Leuven ethics committee. In this online project, participants were presented with cue words and were required to generate three associative responses for each of the cue words separately, for a total of 14 to 18 cue words Although data collection in this project continued till 2018, we only analyzed a subset of the data. Our analysis included data collected from the beginning of data collection (2011) till the end of 2015, when we started working on this project. However, our analysis includes most of the entire data collected, data collected for 10,500 out of 12,000 cue words. Our dataset includes cues and associative responses totalling 10,500 words, generated by a total of 73,256 participants (mean age of 36 years [SD = 16], 61% females). Words were removed if they were non-appropriate words (such as offensive words), non-American English version words, proper nouns, and nonsensical words in English. Furthermore, similar words in plural and singular forms were merged and encoded as singular. This process led to a total of 8,963 words that were included in the final analysis.

### Phonological network estimation

The representation of the phonological layer was based on a method developed to analyze phonological networks based on phonological similarity^36,37^. First, the written words were converted into a computer readable phonological transcription. Next, links were placed between words according to the Levenshtein edit distance of 1—one word differed from the other by either adding, deleting, or substituting only one phoneme^37^. Only pairs of nodes with an edit distance of 1 were retained in the final adjacency matrix, leading to a subset of nodes (4980 words) from the original list of cue words (8963 words). The links between all pairs of cue words defines a symmetric similarity matrix whose (*i*, *j*) element denotes the phonological similarity between words *i* and *j*. This matrix can be studied in terms of an adjacency matrix of an unweighted, undirected network, where each word is a node and a link between two nodes (words) represents a phonological edit distance of one between them. As all edges are unweighted and uniformly equal to one, distance between word pairs in the phonological network can be measured by counting the number of links that separate them.

### Semantic network estimation

The representation of the semantic network was based on a method developed to analyze semantic networks based on free associative responses generated to cue words^38,39^. Specifically, the more similar associative response that are generated to a pair of cue words, and the larger number of participants generating these associative responses to these cue words, the stronger the link between the word pair. The calculation of a link between two words (which represents the semantic similarity between them) was achieved in the following way: For each pair of cue words, we analyzed only the associative responses generated to them (similar to the method used in 1). For each of these matched associative responses, we sum the lower amount of participants generating them: $Link\left({C}_{i},{C}_{j}\right)=\sum _{k=1}^{Associations}\mathrm{m}\mathrm{i}\mathrm{n}(\#P{\_C}_{i}A\left(k\right),\#P\_{C}_{j}A(k))$, where Associations is the total number of associative responses given to cue words *i* and *j*, and #*P*_*C*!/!*A k* is the amount of participants in the sample generating the *k*’th associative response to cue words *i* and *j*. Our method accounts for not only the correlation of associations based on the overlap of associative features to a pair of cue words, but also the number of participants generating these overlapping associative features. In order to filter noisy links that were a result of one association or a result of an overlapping association that was a result of a single participant’s response, the contribution of an overlapping association to the link strength of each pair of cue words was considered only if more than one participant contributed to the overlapping association.

### Network construction

Using free association data generated to the cue words, we constructed a weighted undirected semantic network^39^, where nodes represent these cue words and links are defined by the similarity of responses generated between cue words. From the same cue words used to construct the semantic network, we constructed an unweighted phonological network^36^. The semantic layer is very dense, with its giant component comprised of 8963 nodes and over 12 M links, whereas the phonological layer is very sparse, with its giant component comprised of 4980 nodes and 16,943 links (SI Figs. 1, 2), both from the about 44 M possible links (Fig. 1A). While both layers include these common 4980 nodes, some links connecting these nodes overlap across the layers, and some do not (Fig. 1A). For example, the link between the pair of cue words *incense* and *intense* is overlapping (exists in both layers) while the link between the cue words *intent* and *indent* is non-overlapping, since these two cue words only have a direct link in the phonological layer (Fig. 1B). As such, this defines a multilayer network, unlike a multiplex network in which there is full node alignment across the layers^40^. When combining the two layers into a multilayer, new paths between cue words across the two layers arise. These new paths usually shorten the distance between pairs of nodes in each of the layers separately.

First, we analyzed the overlapping links regions (area I in Fig. 1A), i.e., testing the similarity between the two layers by analyzing the link overlap (Fig. 2). We then tested the interaction between the two layers (area II in Fig. 1A) by analyzing the effects of adding non-overlapping links from one layer to the other (Fig. 3). Finally, we examined the advantage of a multilayer network architecture when compared to single-layer phonological and semantic networks (Figs. 4, 5).

### Phonological-semantic overlapping link analysis

We computed the correlations of links across the two layers^41^. To do so, we calculated the fraction of overlapping links across the two layers and compared it to the fraction of overlapping links in a random network. We take advantage of previously developed methods to compare between links across different layers in multilayered networks^23,42^, adapting these methods to also account for differences in path length of these links.

First, we calculated the overlap of the phonological links within a moving window of increasing semantic link strengths. We sorted the semantic links in an ascending order according to their strength. Then, we divided the semantic links into equal sized windows of 100,000 links. The size of the window was set in a way that allowed a high resolution for weak links and strong links (which are much less common). In order to test the statistical significance of the overlap of the phonological links to that of the semantic links within each semantic strength window, we randomly chose 10,000 semantic links and calculated the fraction of overlapping semantic links and phonological path distances. Additionally, in order to test the strength of the overlap of the phonological network to the semantic network within each semantic window, we assessed the overlap between the phonological network with a random network—with a similar number of nodes and links to the phonological network—and with the semantic network (SI Fig. 3 shows the overlap without the division by the randomly chosen network overlap). Finally, we divided the fraction of phonological-semantic overlap with the fraction of random-semantic overlap. We repeated this process for 100 iterations and calculated the mean overlap value and its standard deviation. We conducted the same analysis for different phonological link distances of one to three and normalized each phonological distance with their number of links.

Second, we measured the fraction of overlapping semantic links from three equally sized groups with different semantic strengths (weak, medium, strong) for a given phonological path distance. For each phonological path distance, we randomly chose 3,000 phonological links and calculated the fraction of overlapping weak, medium, and strong semantic links. We repeated this process for 100 iterations and calculated the mean overlap fraction and its standard deviation. We compared this overlap of a given phonological path distance with a random network. This random network was generated by permutating the semantic links, thus retaining the same number of nodes and edges while removing any original node-link relation.

### Phonological-semantic non-overlapping link analysis

We analyzed the effect of the non-overlapping phonological link (shortest path) distance with different distances on the semantic layer by adding non-overlapping phonological links to the semantic layer, compared to adding randomly chosen links, and measuring the relative path distances that were reduced in the semantic layer. When adding non-overlapping phonological links to the semantic layer, we have to decide how to refer to the strength of the semantic links compared to the phonological links. Thus, we analyzed the path distances that were reduced over a filtered unweighted semantic network, where we kept the top 1% of the semantic links and converted the semantic layer from weighted to unweighted. We also tested the effect on the top 3% and 5% of the semantic links and found similar results (SI Figs. 5, 6). For each of the phonological path distances (from one to six), we randomly chose half of the non-overlapping phonological links (4439 links) and separately added them to the semantic layer. We then randomly chose a similar number of links (4439 links) from the theoretical fully connected semantic network (~ 44 M links), but which do not exist in the empirical data (~ 12 M links) or were not the added non-overlapping phonological links. We repeated this process for 100 iterations and in each iteration calculated the sum of path distances that were reduced.

We calculated the distribution of path distances that were directly reduced, defined as the number of pairs of nodes that their path distance was reduced directly by the added links. We computed this reduced distance effect from any original path distance to path distance of one, and path distances that were indirectly reduced, i.e., reduced path length between a pair of words that is a result of new paths that appeared in the network due to the addition of new links. Then, we calculated the ratio between the sum of path distances that were reduced due to the addition of the non-overlapping phonological paths with different distances on the semantic layer and that of the addition of the randomly chosen links. Finally, we normalized the sum of reduced distances due to adding phonological links in each iteration by the mean sum of reduced path distances of randomly chosen links. Thus, the closer this fraction is to one, the weaker is the effect of adding non-overlapping phonological links of a specific distance. We also calculated the ratio between the number of pairs of nodes where their path distance was reduced directly by the non-overlapping phonological links and those of the randomly chosen links. Thus, we obtained a relative effect of the addition of non-overlapping phonological links on the semantic layer.

Finally, we measured the effect of adding non-overlapping semantic links on the phonological layer. We added non-overlapping semantic links from three different groups of semantic strength (weak, medium, strong) that are maximally separable, and compared their relative effect to randomly chosen links. Here, for each iteration, we chose randomly ~ 800 semantic links from each group (the three groups of semantic strength and random group). The number 800 was chosen in order to maintain a similar proportion of phonological links added to the semantic network (4439/ ~ 100,000 links—the size of the semantic network) and added them to the phonological layer. We then randomly chose a similar number of links from a theoretical fully connected phonological layer that does not exist in the empirical phonological layer (18 K links) and were not the added non-overlapping semantic links. We repeated this process for 100 iterations and in each iteration calculated the sum of path distances that were reduced. Then, we calculated the ratio between the sum of path distances that were reduced due to the addition of the non-overlapping semantic links with different strengths on the phonological layer and the sum of path distances that were reduced due to the addition of the randomly chosen links. We also calculated the ratio between the number of pairs of nodes where their path distance was reduced directly by the semantic links and those of the randomly chosen links (each group separately). Thus, we obtained a relative effect of the addition of non-overlapping semantic links on the phonological layer.

### Phonological-semantic multilayer analysis

We examined the benefit of the multilayer network by comparing multiplex, semantic, phonological, and a random network, consisting of the same set of nodes and same number of links. We compared the average distances of these networks, and how it varies as a function of the strength of semantic links (based on a moving non-overlapping window, from the strongest to the weakest link). To conduct this analysis, first, we filtered the links of the semantic network to the same average degree and to the same number of nodes that overlaps with the phonological network (4980 nodes). We keep the same nodes in both layers by applying a minimal spanning tree, which identifies the minimum number of links that connects this subset of nodes. Next, according to a moving percentile of link strength in the semantic network, where in each step we include different links in the semantic network, we add semantic links until we get to the same number of links (16,943) and average degree (3.5) as in the phonological network. We used a resolution of 0.5 percent—in each step when we include weaker links. This results in two layers with the same mean degree and the same number of links and nodes. In order to compare the distances of the semantic network, which are weighted, and the phonological network, which is unweighted, we transformed each semantic network from weighted to unweighted. In order to create the multiplex semantic-phonological network, we merged the filtered semantic network with the phonological network. Then, in order to get to the same degree, we used a minimal spanning tree approach^43^. The result of this process was a multilayer network with the same set of nodes as the phonological and semantic networks, yet with lower degree. Additionally, at this point we have a non-equal number of links in the semantic and phonological network. To correct these differences in links and average degree, which affects the network distance, we added semantic and phonological links to get to the same number of semantic and phonological links in the multilayer network, and to have the same average degree in the phonological and semantic networks. After constructing a phonological network, semantic networks and multilayer networks (from various semantic strengths), we calculated the average distance of all possible pairs of nodes in each of the networks.

In order to test the effect of the multilayer network structure on overall distances in the network, including the distribution of semantic and phonological links on this semantic network, we applied a community detection analysis using the Louvain community detection method^44^, with Gamma = 1 (Gamma sets the resolution of the community detection method)^44^. We applied the community analysis on the semantic unweighted filtered network (of the top 10% of links). Then, we compared the fraction of inter-community links (inter-links) of the different networks—semantic networks from different strengths, phonological network, and random network with a similar size.

Next, we analyzed the effect of using different proportions of semantic-phonological links on the average distance of the multilayer network. In order to construct a multilayer network with different proportions of semantic and phonological links, we merged the phonological network with the semantic unweighted network (from a specific window of semantic strengths). Using a minimal spanning tree approach, we achieved a specific proportion of semantic and phonological links (this proportion depends on the semantic strength). According to this specific proportion, we added semantic and phonological links until we get to the same degree of the phonological network, with a range of proportions of between 0.2 to 0.76 semantic links. With a resolution of 0.05 of the semantic strength, we measured the average distance of the multiplex network. We applied this analysis on different multilayer networks that were based on three different semantic networks varying in semantic strength (top 1%, 4%, and 7% of strongest links).

Finally, we examined the generality and validity of the multilayer network architecture having optimal path distances by reanalyzing data collected by Kumar, Balota, and Steyvers^32^. In their study, the authors estimated a large-scale semantic network based on the University of South Florida Free Association Norms, generated to a list of 5000 cues^45^. Aiming to replicate and extend a previous study by Kenett et al.^31^, Kumar et al. had participants make relatedness judgments to pairs of cue words that varied in the semantic distance between the words based on the path length in their semantic network for those two words^32^. The authors recorded participants decisions (related vs. non-related) and the reaction time (RT) by the participants to make their decisions. In line with the findings of Kumar et al.^32^ and Kenett et al.^31^ who found a quadratic relation between path length in semantic networks and participants’ RT, we focused our analysis only on path length of 1–4. We examined how our multilayer network architecture relates to the data collected by Kumar et al. in the following way: First, we identified links from the Kumar et al. semantic network that corresponded with our semantic network. Next, to compare the RTs of Kumar et al. network with our semantic and multilayer networks, we constrained our semantic and multilayer networks so that their average degree matches the average degree as in the Kumar et al. network^32^, and degree distribution with high similarity (SI Fig. 7). We do so by applying a spanning tree on the semantic layer (that includes all links with strength larger than 2) to keep shared nodes with the phonological layer. Next, we randomly added semantic links to the semantic layer to achieve the same average degree (11.7) of the semantic network estimated by Kumar et al.^32^. Our multilayer network was constructed by adding phonological and semantic links at a 20%–80% ratio. We repeated this process for five times (constructed from different links at each iteration) and computed the average RT for each path length for each of these types of networks (semantic and multilayer networks) over these five iterations. The semantic network estimated by Kumar et al.^32^, however, has been provided after construction, thus we could not conduct a similar iterative process on it. Therefore, to construct a distribution of semantic networks from this network, we randomly selected 50% of the links from this network and re-iterated this process ten times. Such an approach matched the three types of networks—our semantic and multilayer networks, and the semantic network from Kumar et al.^32^—and allowed us to directly compare the effect of path length in these networks on RT, as collected by Kumar et al.^32^.

## Results

### Phonological-semantic overlapping link analysis

To examine the potential overlap between the two layers, we conducted two complementary analyses: first we examined the tendency of semantic links of different strength to have corresponding phonological links (Fig. 2A). We then examined the tendency of phonological links of different distances to have corresponding semantic links (Fig. 2B). In both analyses, we compared the fraction between the overlapping links in the two layers and random links (random network). The random links were constructed by randomly permutating pairs of phonological/semantic links so that nodes in the semantic/phonological networks retained their original degree, but their links were completely shuffled. The random links were not expected to have any correspondence with the semantic/phonological network.

To conduct our analysis, we first computed the phonological path length between all possible pairs of nodes, according to the shortest distance between them^46^. For example, in the phonological layer the following nodes are connected to each other: *intend* → *intent* → *invent* → *invest* (Fig. 1B). As such, this chain includes the following phonological links between pairs of nodes with varying distances: Phonological link of distance 1—[*intend*, *intent*], [*intent*, *invent*], [*invent*, *invest*]; Phonological link of distance 2—[*intend*, *invent*], [*intent*, *invest*]; Phonological link of distance 3—[*intend*, *invest*]. We conducted this classification process for all possible pairs of nodes in the phonological layer to compute phonological links of all possible distances. Next, we sorted the semantic links in an ascending order according to their link strength. We divide them into equally sized windows of 100,000 semantic links (“Materials and methods”). For every phonological path distance, within each window of semantic links, we calculated the fraction between the number of overlapping phonological links within the semantic links and the number of overlapping shuffled phonological links within the semantic links, for a set of 10,000 random semantic links (out of the 100,000 links), iterated 100 times (Fig. 2A).

To examine the effect of phonological distance on this overlap ratio, we conducted a one-way ANOVA. This analysis revealed a significant main effect of phonological distance, *F*(2, 57) = 22.03, *p* < 0.001, $\eta ^{2}$ = 0.45; for phonological path distances of one to three, the overlap ratio is significantly higher than random (Fig. 2A). For example, for phonological distance of one, the probability of phonological links to overlap with semantic links in the window with the highest semantic strength is four times larger than in its control random network (SI Fig. 3a). Additionally, when measuring the fraction ratio of each phonological distance as a function of semantic strength, we find that for phonological distance of one, stronger semantic links have significantly higher overlap values than those of phonological distance two and three (all *p*’s < 0.001, Fig. 2A). Thus, strong semantic links have a higher probability to have a matching phonological link with short distances.

Next, we examine the inverse question, namely what is the probability that a phonological path of varying distances will have a matching semantic link of varying semantic strength (Fig. 2B). To calculate the fraction of overlapping semantic links with different phonological distances, we divided the semantic links into three equally sized groups (weak, medium, strong; “Materials and methods”). Then, we computed the fraction of overlapping semantic links (from the different groups) with the phonological paths of different distances. From each phonological path distance, we randomly chose 3,000 links and calculated the fraction of overlap between phonological and semantic links from the three different semantic strength groups, compared to the fraction of overlap with links from the random network. We iterated this process 100 times.

To examine the effect of semantic strength on this overlap ratio, we conducted a one-way ANOVA (Fig. 2B). This analysis revealed a significant main effect of semantic strength *F*(3, 2800) = 8192, *p* < 0.001, $\eta ^{2}$ = 0.90: Stronger semantic links have a higher overlap ratio (all *p*’s < 0.001). Our analysis also revealed a significant main effect of phonological distance, *F*(6, 2800) = 2081, *p* < 0.001, $\eta ^{2}$ = 0.82: Shorter phonological distances have a higher overlap ratio (all *p*’s < 0.001). Finally, our analysis revealed a significant interaction effect between phonological distance and semantic strength, *F*(18, 2772) = 1374, *p* < 0.001, $\eta ^{2}$ = 0.90. This interaction stems from differential effects of increasing phonological distance and varying semantic strength on the overlap of phonological-semantic links, compared to a random network: For strong and medium strength semantic links, as phonological distance increases, the overlap with semantic links decreases (all *p*’s < 0.001).

These findings suggest that phonological paths with shorter distances are highly correlated to strong semantic links, indicating that phonologically similar words have higher probability to also have a strong semantic relation (Fig. 2A). Furthermore, strong semantic links have a higher probability to have a short phonological path, a probability that decays with phonological distance (Fig. 2B). For higher phonological path distances, the overlap of strong semantic links becomes relatively closer to randomly chosen links.

### Phonological-semantic non-overlapping link analysis

Next, we examined the relation between the two layers focusing on non-overlapping links. Non-overlapping links occur when two nodes are connected via only a phonological or a semantic link. Since these links exist in one layer of the multiplex and not in the other, they could be independent of each other. To test this, we added non-overlapping links from one layer to the other and measured their effect on reducing the overall distances between nodes in the layer, compared to the effect of adding random links.

First, we add non-overlapping phonological links (of various distances) to the semantic layer and measured their effect on reducing the overall distances between nodes in the semantic layer (Fig. 3A). Since the semantic network is highly connected (having about 12 M links which is over 30% of all possible links), the distances in the semantic network are very short (SI Fig. 1c). Thus, the effect of adding non-overlapping phonological links to the full semantic network is small and difficult to observe. Therefore, we analyzed the effect of adding non-overlapping phonological links on the distances of the semantic layer comprised of the top 1% of the strongest semantic links, which has a broader distribution of distances. Such an effect is compared to adding randomly chosen links (which do not overlap with semantic and phonological links). We iterated this analysis 100 times (Fig. 3A). A one-way ANOVA revealed a significant main effect of phonological distance *F*(5, 594) = 2792.49, *p* < 0.001, $\eta ^{2}$ = 0.96. Phonological links of distance one had a significantly larger effect on reducing distances compared with random links. Additionally, we found a gradual decrease of this effect SD phonological distance increased to six (phonological distance of six is like random), suggesting that the relation of the phonological layer to the semantic layer gradually weakens with phonological distance (all *p*’s < 0.001).

Next, we examined what drives this effect, by investigating which semantic path distances were reduced as a result of adding non-overlapping phonological links, compared to adding randomly chosen links. We calculated the ratio between the number of pairs of nodes in the semantic layer where their path distance was reduced to one, due to adding non-overlapping phonological links (of one to six) and due to the addition of random links (Fig. 3B). A two-way (phonological distance x semantic distance) ANOVA revealed a significant main effect of semantic distance, *F*(4, 2970) = 195.34, *p* < 0.001, $\eta ^{2}$ = 0.21, a significant main effect of phonological distance, *F*(5, 2970) = 4.91, *p* < 0.001, $\eta ^{2}$ = 0.01, and a significant interaction between phonological and semantic distances, *F*(20, 2970) = 10.84, *p* < 0.001, $\eta ^{2}$ = 0.07. We found that for short semantic distances (< 4), adding non-overlapping phonological links significantly reduced more distances than by adding random links (ratio scores higher than one). For semantic distances above four steps, adding non-overlapping phonological links reduced fewer distances than by adding random links (ratio scores lower than one). Furthermore, this effect was stronger for shorter phonological distances (all *p*’s < 0.001). This demonstrates that phonological and semantic links are not independent even within the ensemble of non-overlapping links, which indicates a strong interaction between phonological and semantic layers. To examine the robustness of our analysis, we conducted a similar analysis using the top 2% and top 3% of the strongest semantic links (SI Fig. 4). These additional analyses revealed similar results to those found for the top 1% (SI Figs. 5, 6).

Next, we examined the effects of adding non-overlapping semantic links of varying strength to the phonological layer. We used the same methodology as before and measured the effect of adding non-overlapping semantic links on reducing path distances in the phonological layer, compared to adding randomly chosen links. Since the semantic network is very dense, we compared the effect of adding three well separated groups of semantic links from various strengths (weak, medium, strong; “Materials and methods”) to the effect of adding randomly chosen links. We reiterated this analysis 300 times (Fig. 3C). A one-way ANOVA revealed a significant main effect of semantic strength *F*(2, 897) = 364.564, *p* < 0.001, $\eta ^{2}$ = 0.45; adding non-overlapping semantic links reduced the overall distances in the phonological layer more than by adding random links. We found a gradual effect of semantic links according to their strength on reducing distances compared to random links (all *p*’s < 0.001); strong semantic links have the smallest effect on distance reduction, suggesting that the relation of the semantic layer to the phonological layer is gradually decreasing with decreasing semantic strength.

Finally, we examined what drives this effect by investigating which specific phonological path distances were reduced as a result of adding each of the different groups of semantic links, compared with adding randomly chosen links. This was achieved by calculating the ratio between the number of pairs of nodes in the phonological layer where their path distance was reduced from any path distance to one (due to the addition of the semantic links) and their analogues from adding random links (Fig. 3D). A two-way (semantic distance ×x phonological distance) ANOVA revealed a significant main effect of phonological distance, *F*(5, 5382) = 309.61, *p* < 0.001, $\eta ^{2}$ = 0.22, a significant main effect of semantic distance, *F*(2, 5382) = 38.46, *p* < 0.001, $\eta ^{2}$ = 0.01, and a significant interaction between phonological and semantic distances, *F*(10, 5382) = 43.48, *p* < 0.001, $\eta ^{2}$ = 0.08. We found that strong semantic links reduced shorter phonological distances to one significantly more than random, and that this effect was reduced as semantic strength decreased (all *p*’s < 0.001). For example, the effect of adding strong semantic links in reducing phonological distance of two to one is 1.3 times random, whereas adding medium semantic strength is 1.2. Thus, semantic links tend to affect short phonological path distances; the stronger the semantic link, the stronger this effect is. These findings correspond to our findings on adding non-overlapping phonological links to the semantic layer (Fig. 3A,B), and further demonstrates the strong interaction between the semantic and phonological layers.

### Phonological-semantic multilayer analysis

After analyzing the similarity of the semantic and phonological layers and the interactions of these layers with each other, we examined the potential advantages of combining both layers into a multilayer network on lexical access (“Materials and methods”). To do so, we compared for the *same* common nodes and the *same* number of links, the average distance between pairs of cue words in the semantic network, in the phonological network, and in a combined, multilayer phonological-semantic network (equal number of total links), as a function of semantic strength (Fig. 4A). To this end, we calculated the average distances (Fig. 4A) of the semantic, phonological, multilayer and of a random network from the same nodes with similar degree (for detailed process of how these networks were formed and processed, see “Materials and methods”). We conducted this process from the window of the strongest semantic links all the way down to the window of the 90th percentile of strongest semantic links. At the window of 90th percentile semantic links, the semantic network average distance was indistinguishable from the random network average distance, as well as the multilayer network (Fig. 4A). We illustrate a pairwise comparison of distances in the semantic and phonological pairs of nodes using a heatmap for the top 100% (Fig. 4B) and top 90% (Fig. 4C) of semantic links, which represents the two extreme points in Fig. 4A.

To examine the effect of the networks on their mean distance across the various semantic links strengths, we conducted a one-way ANOVA, which revealed a significant main effect of network, *F*(3, 60) = 59.22, *p* < 0.001, $\eta ^{2}$ = 0.75. This effect is driven by the difference in the mean overall distances in the phonological (5.66), semantic (4.32), multilayer (4.19) and random (4.11) networks (all *p*’s < 0.001). Furthermore, we found an effect of the strength of the semantic links on the mean overall distance of the semantic, multilayer, and random networks, compared to the phonological network: While the average distance (6.98) of the semantic network with the strongest links (100%) was higher than that of the phonological network (5.66**);** when moving to slightly weaker semantic links (99%) the average distance of the semantic network (4.87) becomes smaller than that of the phonological network (5.66). In fact, taking weaker semantic links, of the top 90%, leads to an average distance that is comparable to a random network (Fig. 4A).

To better understand the origin of these results we apply the Louvain community detection method^44^ to examine the structure of the semantic network. This was done in order to test the effect of the network structure on overall distances in the network (“Materials and methods”). We found over 30 communities with between 50 to 600 nodes. Next, we tested where the phonological, semantic, and randomly chosen links tended to appear—within communities (intra-links) or between communities (inter-links). We found that phonological links and weak semantic links, almost as random links, were mostly inter-links (fraction of about 0.96 are interlinks) and, in marked contrast, we found that the very strong semantic links were mainly intra-links (Fig. 4D). Furthermore, the relative fraction of the semantic inter-links increased with the decrease of semantic link strength.

Next, we examined the effect of using different fractions of semantic and phonological links in the multilayer network, while keeping the same number of links. We measured the average distance of the multilayer phonological-semantic network for different percentages of the top semantic links (99%, 96% and 93%) as a function of the fraction of the semantic links (Fig. 4E). We found that there was an optimal fraction of phonological and semantic links in terms of achieving a minimal (optimal) average network distance in the multilayer network: the optimal fraction of semantic links was higher for networks with weaker semantic links, suggesting that the role of phonological links is more vital in multilayer networks with stronger semantic links. Furthermore, we found that the overall optimal distance decreased with the decrease of semantic strengths of the multiplex (Fig. 4E).

Finally, we compared the RTs as a function of network distance of the three networks: the semantic network estimated by Kumar et al., our estimated semantic network, and our multilayer network (Fig. 5). Across all three networks, the RTs showed similar patterns. To compare RT effects for each path length across the three networks, we conducted a Kruskal–Wallis independent samples test. This analysis revealed a significant effect for path length of two, H(2) = 12.59, *p* < 0.001. To examine what drives this significant main effect, we conducted a Mann–Whitney U independent samples test. This analysis revealed a significant difference between the RT of our multilayer network (mean = 756.85 ms, SD = 3.68 ms) and our semantic network (mean = 767.85 ms, SD = 7.93 ms), U = 0, p < 0.001, as well as the Kumar et al. semantic network (mean = 761.94 ms, SD = 1.99 ms), U = 2, *p* < 0.001. Finally, this analysis also found a significant difference between our semantic network and the semantic network of Kumar et al., U = 7, *p* < 0.03. These results suggest that our multilayer network facilitates shorter RT times, which are considered to be related to cognitive effort.

## Discussion

Language is a complex phenomena, involving multiple linguistic compnents, such as syntax, morphology, phonology and semantics^1^. To achieve meaningful linguistic communications, one must access and retrieve information from their ‘mental lexicon’, which stores the representations of concepts. Thus, lexical access is a critical process (albeit just one of many) in human communication, yet the nature of this process remains debated until this day^2,3^. Linguistic theories agree that lexical access includes two components, a phonological and a semantic component. However, these theories propose different relations between these two components^3,5^. One promising approach to empirically investigate the relation between these two components is via applying quantitative tools from network science to study cognition^17^.

While the application of network science to cognitive phneomena^15,17^ has lead to quantitative insights regarding phonology^18,36^ and semantics^19,29^, such insights are confined only to each of these processes separately. In the current study we apply a multilayer network analysis approach to examine the structure and the relation between a phonological layer and a semantic layer, and how this multilayer network structure could be related to lexical access. Multilayer networks have become increasingly popular in studying complex systems with multiple dimensions^20,22^, and have recently been applied to study language development and impairment^23,24,47^. Such multilayer network research currently focuses on how words sound, phonology, and their meaning, semantics. This line of work is only a first step in analyzing linguistic processing as a multidimensional, multilayer network structure. While adding more linguistic layers (such as morphology and syntax) will generalize this quantitative approach to study a broader range of linguistic phenomena, it will also make this analysis more complex. In accordance with this developing line of research, we analyze a large-scale multilayer network that is composed of a phonological layer and a semantic layer. Our linguistic multilayer network is estimated from a unique dataset of free association responses collected in English^28^ based on a Big Data approach, collected over several years. Such a dataset provides us a unique opportunity to represent a subset of the multidimensional structure of the mental lexicon based on behaviourally collected data^29,30^. Such an approach allows us to empirically examine the relation between phonology and semantics in lexical access, a critical component of linguistic processing, but its nature is still debated.

The interactive model of lexical access^2,3,48^ argues that the phonological and semantic attributes of related words are activated automatically due to feedforward and feedbackward processes over a densely connected lexical network during the retrieval process of a particular word^3^. We find here that the phonological and semantic layers are highly similar, and are strongly interactive: The closer two nodes are in the phonological/semantic layer, the higher is the probability that they will also have a similar shorter path in the semantic/phonological layer. These findings highlight how the redundancy between the two layers may increase efficient activation and retrieval of concepts and contextually relevant concepts.

Next, we examine the relation between the two layers by investigating the effect of adding non-overlapping links from one layer to the other. We find that the effect of adding non-overlapping links strongly effects the shortening of path distances between nodes that have short path distances between them. Recent cognitive network studies have illustrated the importance of path distances over cognitive networks both at the phonological^34^ and at the semantic^31^ levels. At the phonological level, shorter phonological distances have been related to both successful retrieval of words and to increased phonological errors^34^. Furthermore, in a phonological association task (generate a word that sounds similar to a cue word), it was found that the majority of the responses (94%) had a distance of two or less from the cue word^34^. At the semantic level, path distance in a semantic network predicted participants performance in judging whether two words were related to each other^31^. Participants judged word pairs as related only for word-pairs with short distances between them (less than 4 steps)^31^. Importantly, these cognitive systems appear to strive for generally short distances while maintaining an overall structure to enhance lexical access, leading to a fragility-efficiency trade-off^49,50^.

Given the importance of shorter path distances for phonological and semantic processing, the reduction effect we find may play a critical role in facilitating such cognitive processing. Thus, the interaction between these two layers might be crucial for allowing more efficient lexical access, by reducing path distances between nodes in a multiplex cognitive network. For example, in the phonological layer, the cue words *intend* and *invest* had a path distance of three (*intend* → *intent* → *invent* → *invest*). However, in the semantic layer these cue words *intend* and *invest* are directly connected (Fig. 1B). Thus, in a multilayer phonological-semantic network, the distance between *intend* and *invest* is much shorter than in a phonological only network, enhancing the lexicon’s efficiency in lexical access even in potential impairments^26,51^.

We examined the advantage of a linguistic multilayer network architecture on language processing, compared to a single layer phonological, semantic, and random networks with the same number of links. We find that the multilayer network has a significantly shorter average distance than the phonological and semantic layers separately, thus providing empirical support for the benefit of a multilayer network for lexical access. Furthermore, we find that decreasing the semantic strength of the links that compose the semantic network has a drastic effect on reducing the distance in the network, the community structure of the network, and the optimal proportion between phonological and semantic links in the multilayer network architecture. We also find that for the strongest semantic links (top 100% percentile), the phonological network is more efficient than the semantic links, the semantic network is mostly comprised of intra-links, and a major reliance on phonological links optimizes the multilayer network. However, when including weaker semantic links (top 95% percentile), the semantic network has an average distance that is already similar to that of the multilayer network, the community structure of the semantic network is mostly inter-links, and a major reliance on weak semantic links optimizes the multilayer network architecture. This effect demonstrates the role of weaker semantic links in increasing linguistic network efficiency^52^. Our findings are in line with previous studies that highlight the role of weak semantic links in creative thinking in semantic network^39,53^ and multilayer^54^ networks. Furthermore, our findings may indicate that higher creative individuals can be less dependent on phonological processing for lexical access and that utilizing the phonological layer is most effective for people who rely less on weak semantic links.

Overall, our findings provide empirical evidence supporting the processes theorized by the interactive model of lexical access^3^: both phonological and semantic layers are highly similar, but potential interaction between these layers in a multilayer network lexicon architecture facilitates processing efficiency by reducing overall distances between concepts in a multiplex mental lexicon. To support this claim, we reanalyzed data collected by Kumar et al.^32^, who replicated and extended previous work of Kenett et al.^31^ showing the effect of path length in a semantic network on participants’ performance in judging whether word pairs were related to each other. These two studies aim to directly link semantic path length to the process of semantic priming—a quicker decision to judge that a target word is related to a prime word when these two words are conceptually related^55^—by showing that as semantic path length increases, it becomes harder (e.g., takes more time and variability in decisions increases) to make such decisions. While additional non-facilitative semantic processes can affect participants’ response time in making such decisions^56^, it has been shown that semantic priming facilitates shorter response times^55^. Thus, our rationale for the reanalysis of the response times collected by Kumar et al.^32^ in making relatedness judgments to word pairs with varying path length is the following: if the multilayer network architecture is optimal in lexical access due to a reduced distances effect, than links in our multilayer network should capture shorter response times in the Kumar et al. data. Indeed, this is what we find—path length of two in our multilayer network leads to shorter response times than those by the similar path length of the semantic network estimated by Kumar et al., as well as our single layer semantic network. While one needs to be cautions in interpreting response times^55^, and our networks only partially overlapped with the semantic network of Kumar et al., this result supports and strengthens our claim that a semantic-phonological multilayer network is optimal in facilitating lexical access due to its reduced distances caused by the integration of phonological and semantic links. Further empirical research is needed—based on the work of Kenett et al.^31^ and Kumar et al.^32^—with multilayer network distances to better directly and empirically examine such beneficial effects of the multilayer network architecture. Finally, weaker semantic links effect the structure and efficiency of the semantic layer, which directly impacts the multilayer network architecture. Our findings generate novel and unique predictions for differential reliance on phonological versus semantic processing, and how this reliance may relate to individual differences in higher-level cognition. Thus, our findings further elucidate the nature of the lexical access process, an essential aspect of human communication.

A few methodological considerations must be considered. First, the methods we used to represent the networks lead to a weighted semantic layer and an unweighted phonological layer. The difference in these two networks—weighted and unweighted—provides methodological challenges in considerations for how to merge these types of networks together. Future research is needed to better match across methods for network representations across different layers (as well as incorporating directed and undirected networks, to account for asymmetrical linguistic phenomena), further elucidating the complexity of human language and efficient communication.

Next, the phonological network was constructed using the Levenshtein edit distance of 1^37^. Links were placed between words that differed from each other by either adding, deleting, or substituting only one phoneme. While this method has been widely applied to represent phonological networks^18^, future research should examine alternative methods to compute phonological links, with higher (i.e., weighted) edit distances or via a phonological association task^34^. In addition, this method for estimating phonological networks leads to a strong core-periphery phonological network structure, with many disconnected phonological nodes, or “isolates”^36^. Such a phonological network structure may introduce noise into our analysis. However, we only include the largest (giant) connected phonological component in our analysis, and we find a strong effect of adding semantic links to the phonological layer. Further studies are needed to assess the potential effect of such a phonological core-periphery structure on cognitive multilayer network architecture. Related to this potential issue, when participants generate free associations, these associations may also be based on phonological, and not only semantic relations. Such phonological-based associative responses introduces noise into how our semantic network is estimated, and potentially inflates the relation between our semantic and phonological layers. However, estimating semantic networks based on free associations is an established and widely used approach^17,19^. In addition, we filter out idiosyncratic associative responses (responses that were generated by less than two participants), to control for such potential noise. Future research should replicate our work using different approaches to estimate semantic networks, such as based on semantic fluency or relatedness judgment responses^17^.

A further limitation is that we interpret our findings of the benefits of reduced distances in a cognitive multilayer network architecture in facilitating communication efficiency, without directly testing for such dynamics. However, current cognitive network research is only recently starting to uncover and examine such cognitive dynamics, based on similar methods to those we applied in our work^17,57^. Much more research is needed to advance our understanding of such cognitive dynamics over memory and linguistic networks, making such work outside the scope of our current study. Future research is needed to start simulating activation processes over a cognitive multilayer network architecture (e.g.,^58^). However, our empirical findings that the multilayer network leads to shorter RTs, based on the data collected by Kumar et al.^32^ strengthens our interpretation of our findings.

Finally, we only examined a cognitive multilayer network that has two layers—a phonological and a semantic layer. Future research is needed to extend our findings in a higher dimensional cognitive multilayer network, that includes further components of language processing, such as morphology, syntax and orthography^24^. Such a higher dimensional cognitive multilayer network could directly lead to a more complete model of lexical access.

In conclusion, in the current study we apply a multilayer network approach to directly study the relations between phonology and semantics in lexical access, a core process required for efficient and meaningful human communication^3^. Our findings provide evidence for an interaction effect between the phonological and semantic processes and provide quantitative findings on the nature of this interactive relation. Thus, the application of multiplex networks in cognition can push the boundaries forward in directly examining cognitive theories of multidimensional systems, such as language.

## Supplementary Information

Supplementary Information.

## References

[1] Fromkin V, Rodman R, Hyams N (2018). An Introduction to Language.

[2] Dell GS, O'Seaghdha PG (1992). Stages of lexical access in language production. Cognition.

[3] Dell GS, Nozari N, Oppenheim GM, Goldrick M, Ferreira VS, Miozzo M (2014). Word Production: Behavioral and Computational Considerations. The Oxford Handbook of Language Production.

[4] Nadeau SE (2012). The Neural Architecture of Grammar.

[5] Levelt WJM (1993). Speaking: From Intention to Articulation.

[6] O’seaghdha PG, Marin JW (1997). Mediated semantic-phonological priming: Calling distant relatives. J. Mem. Lang..

[7] Farrar WT, van Orden GC, Hamouz V (2001). When SOFA primes TOUCH: Interdependence of spelling, sound, and meaning in “semantically mediated” phonological priming. Mem. Cognit..

[8] Jared D, Seidenberg MS (1991). Does word identification proceed from spelling to sound to meaning?. J. Exp. Psychol. Gen..

[9] Watson JM, Balota DA, Sergent-Marshall SD (2001). Semantic, phonological, and hybrid veridical and false memories in healthy older adults and in individuals with dementia of the Alzheimer type. Neuropsychology.

[10] Finley JR, Sungkhasettee VW, Roediger HL, Balota DA (2017). Relative contributions of semantic and phonological associates to over-additive false recall in hybrid DRM lists. J. Mem. Lang..

[11] Hutchison KA, Meade ML, Williams NS, Manley KD, McNabb JC (2018). How do associative and phonemic overlap interact to boost illusory recollection?. Memory.

[12] Watson JM, Balota DA, Roediger HL (2003). Creating false memories with hybrid lists of semantic and phonological associates: Over-additive false memories produced by converging associative networks. J. Mem. Lang..

[13] Amenta S, Marelli M, Sulpizio S (2017). From sound to meaning: Phonology-to-Semantics mapping in visual word recognition. Psychon. Bull. Rev..

[14] Barabási A-L (2016). Network Science.

[15] Baronchelli A, Ferrer-i-Cancho R, Pastor-Satorras R, Chater N, Christiansen MH (2013). Networks in cognitive science. Trends Cogn. Sci..

[16] Karuza EA, Thompson-Schill SL, Bassett DS (2016). Local patterns to global architectures: Influences of network topology on human learning. Trends Cogn. Sci..

[17] Siew CSQ, Wulff DU, Beckage NM, Kenett YN (2019). Cognitive network science: A review of research on cognition through the lens of network representations, processes, and dynamics. Complexity.

[18] Vitevitch MS, Castro N (2015). Using network science in the language sciences and clinic. Int. J. Speech Lang. Pathol..

[19] Borge-Holthoefer J, Arenas A (2010). Semantic networks: Structure and dynamics. Entropy.

[20] Boccaletti S (2014). The structure and dynamics of multilayer networks. Phys. Rep..

[21] Battiston F, Nicosia V, Latora V (2014). Structural measures for multiplex networks. Phys. Rev. E.

[22] D’Agostino G, Scala A (2016). Networks of Networks: The Last Frontier of Complexity.

[23] Stella M, Beckage NM, Brede M (2017). Multiplex lexical networks reveal patterns in early word acquisition in children. Sci. Rep..

[24] Stella M, Beckage NM, Brede M, De Domenico M (2018). Multiplex model of mental lexicon reveals explosive learning in humans. Sci. Rep..

[25] Stella M, Brede M (2015). Patterns in the English language: Phonological networks, percolation and assembly models. J. Stat. Mech Theory Exp..

[26] Castro N, Stella M (2019). The multiplex structure of the mental lexicon influences picture naming in people with aphasia. J. Compl. Netw..

[27] Stella M (2019). Modelling early word acquisition through multiplex lexical networks and machine learning. Big Data Cogn. Comput..

[28] De Deyne S, Navarro DJ, Perfors A, Brysbaert M, Storms G (2019). The, “Small World of Words” English word association norms for over 12,000 cue words. Behav. Res. Methods.

[29] Kumar AA (2021). Semantic memory: A review of methods, models, and current challenges. Psychon. Bull. Rev..

[30] Kenett YN (2019). What can quantitative measures of semantic distance tell us about creativity?. Curr. Opin. Behav. Sci..

[31] Kenett YN, Levi E, Anaki D, Faust M (2017). The semantic distance task: Quantifying semantic distance with semantic network path length. J. Exp. Psychol. Learn. Mem. Cogn..

[32] Kumar AA, Balota DA, Steyvers M (2020). Distant connectivity and multiple-step priming in large-scale semantic networks. J. Exp. Psychol. Learn. Mem. Cogn..

[33] Vitevitch MS, Chan KY, Goldstein R (2014). Insights into failed lexical retrieval from network science. Cogn. Psychol..

[34] Vitevitch, M. S., Goldstein, R. & Johnson, E. In *Towards a Theoretical Framework for Analyzing Complex Linguistic Networks Understanding Complex Systems* (eds Mehler, A. *et al.*) Ch. 2, 29–45 (Springer, 2016).

[35] De Deyne S, Navarro DJ, Storms G (2013). Better explanations of lexical and semantic cognition using networks derived from continued rather than single-word associations. Behav. Res. Methods.

[36] Vitevitch MS (2008). What can graph theory tell us about word learning and lexical retrieval?. J. Speech Lang. Hear. Res..

[37] Luce PA, Pisoni DB (1998). Recognizing spoken words: The neighborhood activation model. Ear Hear..

[38] Kenett YN, Anaki D, Faust M (2014). Investigating the structure of semantic networks in low and high creative persons. Front. Hum. Neurosci..

[39] Kenett YN (2018). Flexibility of thought in high creative individuals represented by percolation analysis. Proc. Natl. Acad. Sci..

[40] Kivelä M (2014). Multilayer networks. J. Compl. Netw..

[41] Nicosia V, Latora V (2015). Measuring and modeling correlations in multiplex networks. Phys. Rev. E.

[42] Gemmetto V, Garlaschelli D (2015). Multiplexity versus correlation: The role of local constraints in real multiplexes. Sci. Rep..

[43] Tewarie P, van Dellen E, Hillebrand A, Stam CJ (2015). The minimum spanning tree: An unbiased method for brain network analysis. Neuroimage.

[44] Blondel VD, Guillaume J-L, Lambiotte R, Lefebvre E (2008). Fast unfolding of communities in large networks. J. Stat. Mech Theory Exp..

[45] Nelson DL, McEvoy CL, Schreiber TA (2004). The University of South Florida free association, rhyme, and word fragment norms. Behav. Res. Methods Instrum. Comput..

[46] Strang A, Haynes O, Cahill ND, Narayan DA (2018). Generalized relationships between characteristic path length, efficiency, clustering coefficients, and density. Soc. Netw. Anal. Min..

[47] Castro N, Stella M (2018). The Multiplex Structure of the Mental Lexicon Influences Picture Naming in People with Aphasia.

[48] Dell GS, Schwartz MF, Martin N, Saffran EM, Gagnon DA (1997). Lexical access in aphasic and nonaphasic speakers. Psychol. Rev..

[49] Faust M, Kenett YN (2014). Rigidity, chaos and integration: Hemispheric interaction and individual differences in metaphor comprehension. Front. Hum. Neurosci..

[50] Pasqualetti F, Zhao S, Favaretto C, Zampieri S (2020). Fragility limits performance in complex networks. Sci. Rep..

[51] Stella M (2020). Multiplex networks quantify robustness of the mental lexicon to catastrophic concept failures, aphasic degradation and ageing. Phys. A.

[52] Latora V, Marchiori M (2001). Efficient behavior of small-world networks. Phys. Rev. Lett..

[53] Kenett YN, Faust M (2019). A semantic network cartography of the creative mind. Trends Cogn. Sci..

[54] Stella M, Kenett YN (2019). Viability in multiplex lexical networks and machine learning characterizes human creativity. Big Data Cogn. Comput..

[55] Balota DA, Yap MJ, Cortese MJ, Watson JM (2008). Beyond mean response latency: Response time distributional analyses of semantic priming. J. Mem. Lang..

[56] Rose SB, Aristei S, Melinger A, AbdelRahman R (2019). The closer they are, the more they interfere: Semantic similarity of word distractors increases competition in language production. J. Exp. Psychol..

[57] Castro N, Siew CSQ (2020). Contributions of modern network science to the cognitive sciences: Revisiting research spirals of representation and process. Proc. R. Soc. A.

[58] Siew CSQ (2019). spreadr: An R package to simulate spreading activation in a network. Behav. Res. Methods.

