# Supplementary material for: Unveiling the nature of interaction between semantics and phonology in lexical access based on multilayer networks
Source: Sci Rep. 2021 Jul 14;11:14479. doi: 10.1038/s41598-021-93925-y (PMC8280146; doi:10.1038/s41598-021-93925-y)
Supplement: Supplementary file 1 — Supplementary Information. [file 41598_2021_93925_MOESM1_ESM.docx]

**Unveiling the nature of interaction between semantics and phonology in lexical access based on multilayer networks**

Orr Levy^1*^, Yoed N. Kenett^2*^, Orr Oxenberg^1^, Nichol Castro^3^, Simon De Deyne^4^, Michael S. Vitevitch^5^ & Shlomo Havlin^1^

^1^ Department of Physics, Bar-Ilan University, Ramat-Gan 52900, Israel

^2^ William Davidson Faculty of Industrial Engineering and Management, Technion—Israel Institute of Technology, Israel

^3^ Department of Communicative Disorders and Sciences, University at Buffalo, Buffalo, NY 14214, USA

^4^ School of Psychological Sciences, University of Melbourne, 2010 VIC Australia

^5^ Department of Psychology, University of Kansas, Lawrence, KS 66045, USA

* Authors contributed equally to the manuscript

**Supplementary Information**

**SI Figure 1**


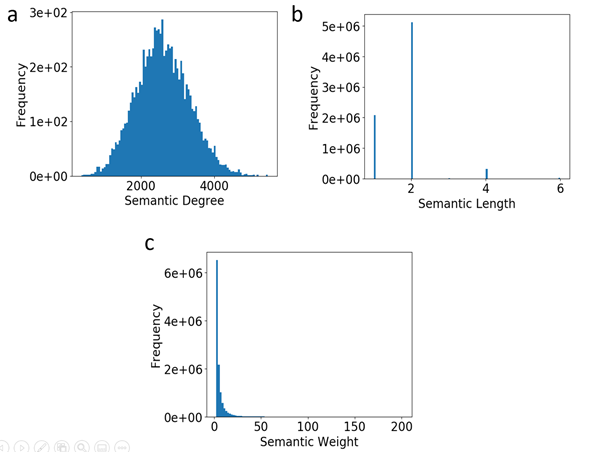


5X10^6

4X10^6

3X10^6

2X10^6

1X10^6

0X10^0

2

4

6

Semantic Weight

3X10^2

2X10^2

1X10^2

0X10^0

2000

4000

Semantic Weight

6X10^6

4X10^6

2X10^6

0X10^6

Semantic Weight

50

100

150

200

Frequency

Frequency

Frequency

SI Fig. 1. **Properties of the semantic network**. (A) Distribution of link degree (B) Distance distribution of semantic unweighted network (C) Degree distribution of nodes

**SI Figure 2**


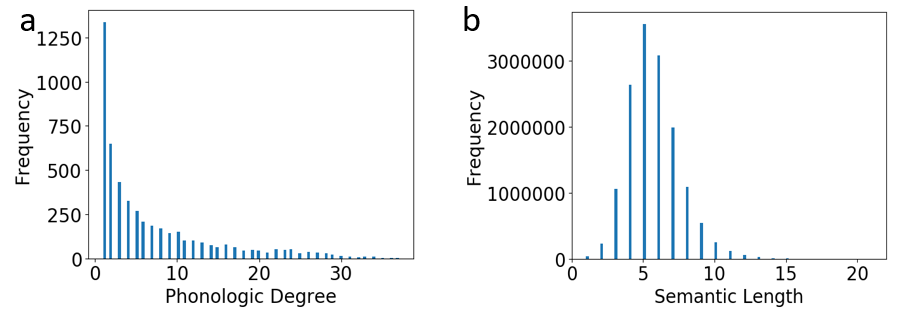


Phonologic Degree

1250

1000

750

500

250

Frequency

Phonologic Degree

Frequency

3X10^6

2X10^6

1X10^6

a

b

**SI Fig. 2. Properties of phonologic network.** (A) Degree distribution of nodes (B) Distance distribution of unweighted network

**SI Figure 3**

c
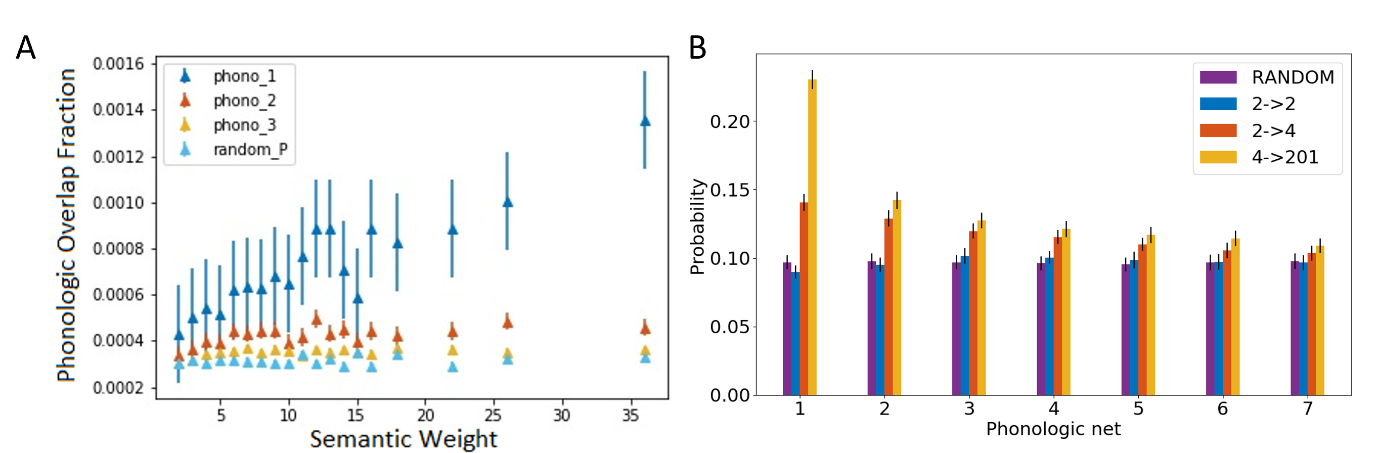


SI Fig. **3. Phonological and semantic overlapping links analysis with different semantic links division**. This analysis is similar to that shown in Fig. 2 with a different weighting of the semantic groups, the three groups of semantic weights are from different strengths. (A) The fraction of overlapping phonological links and semantic links within semantic links, as a function of semantic strength, for different phonological paths distances. Each point represents the ratio of the *average* number of overlapping phonological links divided by the number of the links in the semantic window (bars denote standard error). These averages were obtained from 100 iterations per window. (B) Fraction of overlapping semantic links of different strength with phonological links of different phonological distances. The fraction is with respect to the phonological network. For each phonological path distance, we compare the average fraction of overlapping groups of semantic links of different strength – strong, medium, weak links and shuffled semantic links with phonological links (bars denote standard error). These averages were obtained from 100 iterations per group. Note that for phonological path distance one, the fraction of overlapping phonological with all semantic strengths is close to 0.5.

**SI Figure 4**


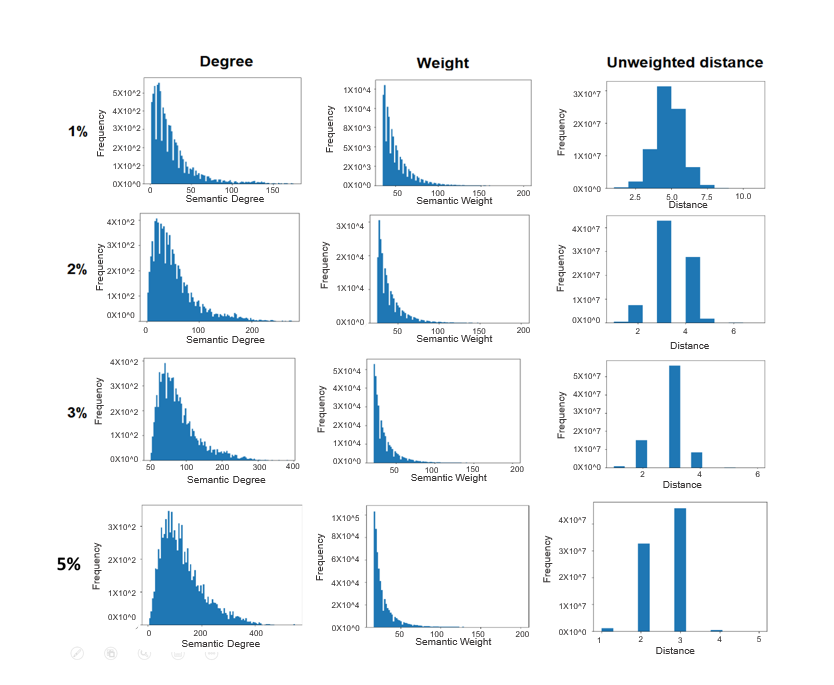


**SI Fig. 4. Properties of top 1%, 2%, 3% and 5% semantic networks.** For each type of top percent semantic network, we measured the degree distribution (left), link weight distribution (center) and its unweighted network distance distribution (right).

**SI Figure 5**


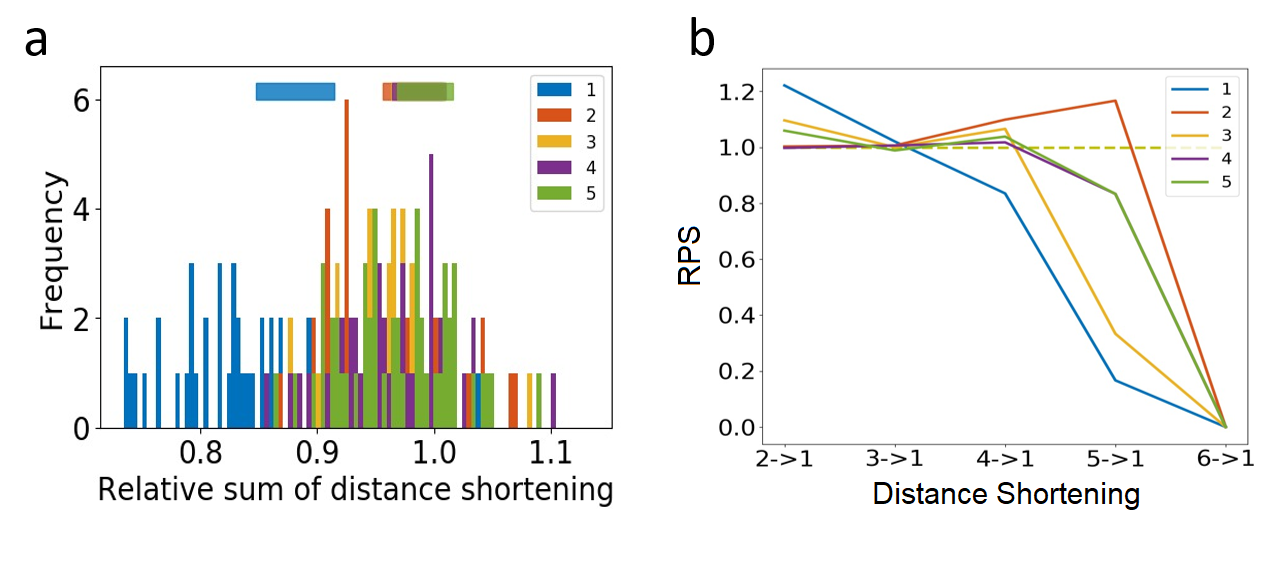


**SI Fig. 5**. **The effect of adding non-overlapping phonological links to the top 3% semantic layer.** (a) The ratio between the sum of distances that are reduced in the semantic layer by adding non-overlapping phonological links of different distances, compared to adding randomly chosen links (adding random links corresponds to 1.00 in the x-axis). The distribution is shown for 50 realizations. The rectangles are colored according to the phonologic path distance represents the mean and the STD of the distributions. (b) Ratio between the number of semantic path distances that are reduced to a distance of one due to adding non-overlapping phonological links and the analogues for adding random links. The x-axis is the semantic path distance that was reduced. In this given example we consider the top 3% of the strongest links in the semantic network. In order to reduce the code runtime, we sampled randomly 400 nodes in the network and calculated this reduction in distance. Y-axis: RPS – Ratio of Path Shortening

**SI Figure 6**


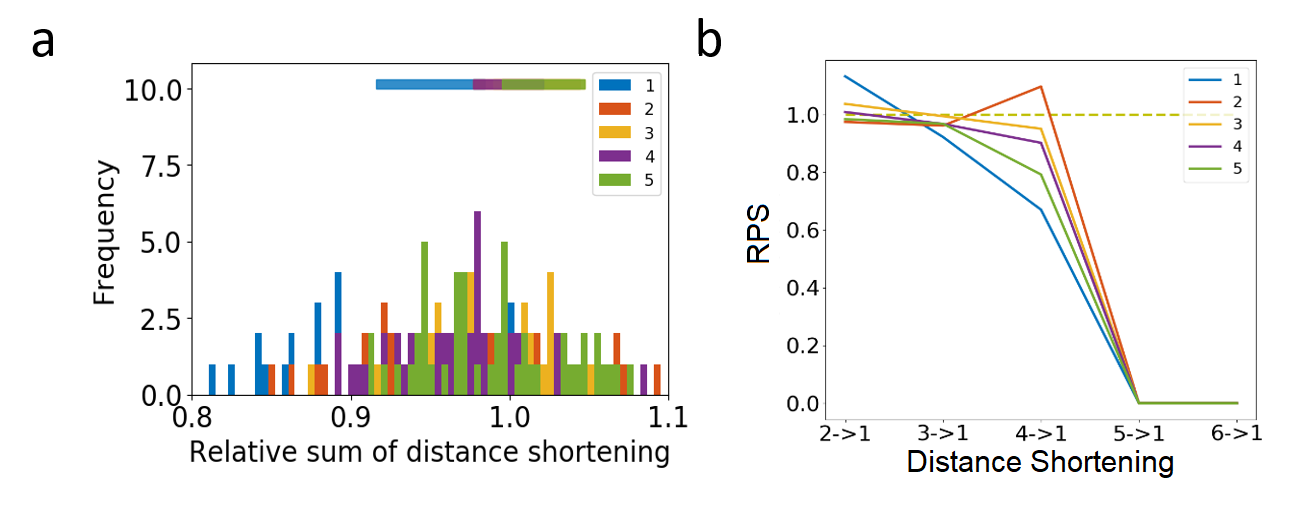


**SI Fig. 6**. **The effect of adding non-overlapping phonological links to the top 5% semantic layer.** (a) The ratio between the sum of distances that are reduced in the semantic layer by adding non-overlapping phonological links of different distances, compared to adding randomly chosen links (adding random links corresponds to 1.00 in the x-axis). The distribution is shown for 50 realizations. The rectangles are colored according to the phonologic path distance represents the mean and the STD of the distributions. (b) Ratio between the number of semantic path distances that are reduced to a distance of one due to adding non-overlapping phonological links and the analogues for adding random links. The x-axis is the semantic path distance that was reduced. In this given example we consider the top 5% of the strongest links in the semantic network. In order to reduce the code runtime, we sampled randomly 400 nodes in the network and calculated the distances reduction.

**SI Figure 7**


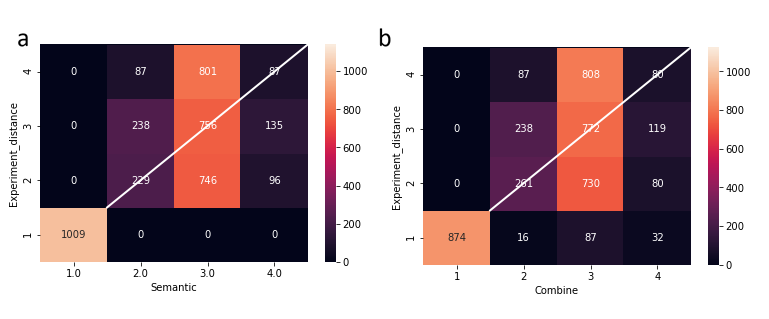


**SI Fig. 7**. **Average distance scatter plots of experimental data and semantic data and combined networks of experiment.** (a) The scatter plot shows the number of pairs of nodes in the network from Kumar, Balota, and Steyvers ^1^, and from the semantic network, according to the path length . (b) The scatter plot shows the number of pairs of nodes in the network from Kumar, Balota, and Steyvers ^1^, and from our multilayer network. The values of the scatter plot represent the number of pairs of nodes who have matching distances where in (a) the X-axis is our semantic network, in (b) the X-axis is our multilayer network and the Y-axis in both (a) and (b) isthe semantic network from Kumar, Balota, and Steyvers ^1^. We use the same set of key words in all networks, and we find that the two distributions share similar characteristics. The scale is from 0 pairs to ~1000 pairs.

1 Kumar, A. A., Balota, D. A. & Steyvers, M. Distant connectivity and multiple-step priming in large-scale semantic networks. *Journal of Experimental Psychology: Learning, Memory, and Cognition*, doi:10.1037/xlm0000793 (2019).
